# An Unusual Presentation of Immunoglobulin G4-Related Disease (IgG4-RD) Causing Subglottic Stenosis

**DOI:** 10.7759/cureus.26250

**Published:** 2022-06-23

**Authors:** Prakash Nallani, Weixia Guo, Ross M Mayerhoff, Alireza Meysami

**Affiliations:** 1 Rheumatology, Henry Ford Health System, Detroit, USA; 2 Rheumatology, Wayne State University Detroit Medical Center, Detroit, USA; 3 Rheumatology, Trinity Health Integrated Healthcare Association (IHA) Medical Group, Livonia, USA; 4 Otolaryngology, Head and Neck Surgery, Henry Ford Health System, Detroit, USA

**Keywords:** rituximab, airway obstruction, glucocorticoids, subglottic stenosis, igg4-related disease

## Abstract

Immunoglobulin G4-related disease (IgG4-RD) is an immune-mediated fibroinflammatory condition that is known to involve multiple organs and was first described as an entity in 2003. It is characterized by lesions with a dense lymphoplasmacytic infiltrate, IgG4-positive plasma cells, storiform fibrosis, and frequently elevated serum IgG4 levels. Organs that are commonly involved include the pancreas, biliary tree, salivary glands, periorbital tissues, kidneys, retroperitoneum, lungs, pleura, thyroid, aorta, and lymph nodes. Rarer manifestations of IgG4-RD include central nervous system (CNS) involvement, prostatitis, mastitis, midline destructive disease, and nasopharyngeal disease. In this report, we discuss an atypical case of a young woman with laryngeal subglottic involvement leading to stenosis and airway obstruction, which was ultimately successfully managed with systemic immunosuppression.

## Introduction

Immunoglobulin G4-related disease (IgG4-RD) is an immune-mediated condition that causes fibroinflammatory lesions, which can affect multiple organs and was first identified as a distinct entity in 2003 [1]. It is characterized by lesions with a dense lymphoplasmacytic infiltrate, IgG4-positive plasma cells, storiform fibrosis, obliterative phlebitis, and frequent elevation of serum IgG4 levels. The most commonly affected organs include the pancreas, biliary tree, major salivary glands, the orbits, and lacrimal glands, kidneys, aorta, retroperitoneum, lungs, pleura, thyroid, and lymph nodes. 

Rarer manifestations of IgG4-RD include CNS involvement, prostatitis, mastitis, midline destructive disease (affecting the nose, paranasal sinuses, and oral cavity), and nasopharyngeal disease. The condition can mimic malignancy, infection, and other inflammatory diseases, including ANCA-associated vasculitis and Sjogren's syndrome, and it is not uncommon for it to be misdiagnosed initially [2]. Primary laryngeal involvement of IgG4-RD is a rare phenomenon, although the head and neck manifestations are commonly seen by otolaryngologists [6]. If left untreated, IgG4-RD can progress to organ dysfunction, organ damage, and even death. 

Here, we describe a case of a young woman presenting with laryngeal subglottic involvement leading to stenosis and airway obstruction. It is important to closely follow individuals with suspected IgG4-RD, often with a multidisciplinary approach, because the formal diagnosis can be delayed if tissue sampling is a challenge. 

## Case presentation

This patient is a 30-year-old woman who presented to laryngology clinic in 2017 after she was unable to be intubated for elective sinonasal surgery at an outside facility due to subglottic stenosis. Prior to that initial visit, she had been experiencing shortness of breath over the past year and was responsive to a few courses of oral glucocorticoids as well as montelukast. Upon presentation to laryngology clinic, the patient complained of nasal obstruction, crusting, and nosebleeds in addition to difficulty in breathing. Flexible laryngoscopy revealed subglottic stenosis. Rheumatology evaluation was requested due to this constellation of symptoms and a family history of autoimmune disease. Initial rheumatologic differential diagnoses included ANCA-associated vasculitis, IgG4-related disease, sarcoidosis, and relapsing polychondritis. Laboratory workup revealed slightly elevated IgG4 subclass at 151 mg/dL (normal: 4-86 mg/dL), slightly elevated ALT but was otherwise normal, including basic metabolic panel, complete blood count, and serum protein electrophoresis (Table 1).

**Table 1:** Relevant Laboratory Test Results GFR: Glomerular filtration rate, AST: Aspartate aminotransferase, ALT: Alanine transaminase, WBC: White blood cells, RBC: Red blood cells

	Result	Normal Value
Creatinine	0.52	<1.16 mg/dL
GFR	>120	>60 ml/min/1.73m2
AST	25	<35 IU/L
ALT	64	<52 IU/L
Alkaline Phosphatase	86	0 - 100 IU/L
Bilirubin, Total	0.5	<1.2 mg/dL
Protein, Total, Serum	6.9	6.0 - 8.3 g/dL
WBC Count	7.3	3.8 - 10.6 K/uL
RBC Count	4.66	4.15 - 5.55 M/uL
Hemoglobin	14	12.0 - 15.0 g/dL
Platelet Count	222	150 - 450 K/uL
Sedimentation rate	9 mm/hr	<20 mm/Hr
C-Reactive Protein	0.4 mg/dL	<0.5
ANA titer	Negative	
RF	<10	<15 U/ml
Cyclic Citr Pep,IgG	0.6	<7 IU/mL
C-ANCA	<1:20	<1:20 Titer
P-ANCA	<1:20	<1:20 Titer
Anti-Ro/SSA antibody	3	0-99
anti-La/SSB antibody	5	0-99
DNA Antibody Screen	Negative	
RNP Antibody	3	<20 UNITS
Smith Antibody	5	<20 UNITS
IgG 1	540	382 - 929 mg/dL
IgG 2	448	242 - 700 mg/dL
IgG 3	54	22 - 176 mg/dL
IgG 4	151	4 - 86 mg/dL
C3 Complement	144	90 - 230 mg/dL
C4 Complement	36	10 - 51 mg/dL
IgA, serum	216	70 - 400 mg/dL
IgM, serum	116	40 - 230 mg/dL
IgG, serum	1,188	700 - 1,600 mg/dL

She had a CT of the chest, abdomen, and pelvis, which demonstrated a 7 mm subpleural left upper lobe nodule with calcification, which was thought to be secondary to prior granulomatous disease. There were no other findings to suggest antineutrophil cytoplasmic antibodies (ANCA) vasculitis or other organ involvement of IgG4-RD. Her first endoscopic airway operation (direct microlaryngoscopy and bronchoscopy with biopsy, radial incisions, dilation, triamcinolone injection, mitomycin c application) provided relief. Pathology of the laryngeal and subglottic tissues showed a small focus of chronic inflammation with stromal fibrosis/hyalinization and a few IgG4 cells present. Due to the limited size of the sample and only the presence of a small number of IgG4 cells, the findings were not conclusive for IgG4-RD. She underwent subsequent endoscopic procedures six months and again 17 months later. In Feb 2018, she underwent a repeat direct microlaryngoscopy with steroid injection due to recurrent respiratory symptoms, after which she had mild improvement. A repeat subglottic tissue biopsy was negative for IgG4 staining. In a discussion between the patient, her otolaryngologist, and rheumatologist, a decision was made to not start her on any systemic therapy. In the meantime, she was also found to have microhematuria. A cystoscopy done in October 2017 was unremarkable. She was then seen by nephrology, who found a few dysmorphic RBCs on urinalysis and proceeded with a kidney biopsy in March 2018, which was normal and negative for IgG4 staining. 

**Figure 1:**
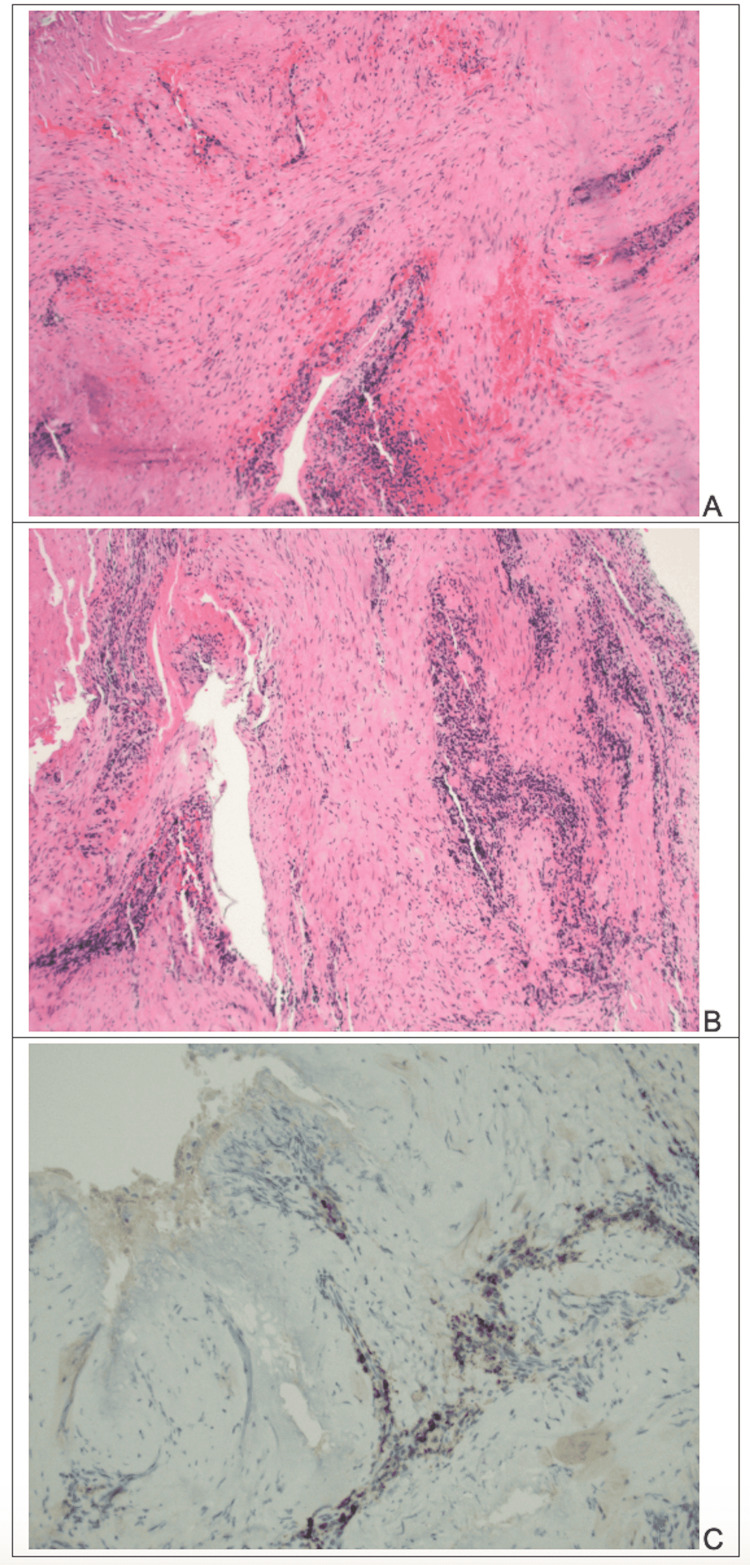
Excisional biopsy of subglottic stenosis A) Respiratory mucosa with Storiform Fibrosis, H&E stain, 10X B) Chronic inflammation, H&E stain, 10X C) IgG4 immunostain demonstrating an increased number of IgG4 cells, 20X

It was noted on a 2019 CT abdomen/pelvis that she had some urothelial thickening of the renal collecting system, which could be due to retroperitoneal fibrosis, which can be seen in the setting of IgG-RD. She then had repeat CT imaging of the chest, abdomen, and pelvis in July 2020, which revealed some scattered benign-appearing pulmonary nodules, but was otherwise unremarkable. She was then treated with serial awake, office intralesional steroid injections. Despite some improvement, her symptoms recurred, along with 60% stenosis. At that time, it was decided that the patient should begin systemic glucocorticoid therapy because she responded to local treatment for a shorter period before recurrence compared to other patients, which suggested the possibility of systemic disease. She was started on a taper of prednisone beginning at 40mg; however, she could not tolerate higher doses of prednisone due to side effects of irritability and insomnia; therefore, a reduced dose of 20mg daily was given for two months. After a follow-up visit with rheumatology, she was increased to 40mg daily for a month. This led to improvement in both nasal and laryngotracheal symptoms. A CT scan of her neck at that time showed left eccentric submucosal thickening of the proximal trachea, 22mm below the level of the glottis and centered just below the cricoid ring. In October 2020, she underwent a repeat microlaryngoscopy and bronchoscopy with a larger excisional biopsy. This time, the biopsy showed dense subepithelial storiform fibrosis and chronic inflammation with increased IgG4 positive plasma cells (33 per high power field at 40x) with less than 50% IgG4/IgG (Figure 1). These findings made the diagnosis of IgG4-RD more definitive based on the 2019 ACR/EULAR classification criteria for IgG4-RD [2]. Rituximab was recommended as steroid-sparing treatment at this time, as she could not tolerate steroids due to side effects. Rituximab was given at 1 gram 14 days apart for two doses. After the first rituximab treatment, she was noted to have bacterial laryngitis that responded to antibiotics. After two weeks of laryngitis treatment and endoscopic assessment, she had improvement in hoarseness and had no dyspnea, dysphagia, and sore throat. Scope revealed significantly improved subglottic stenosis. Since her rituximab infusion, she had not required additional local interventions or injections for 7 months, when she underwent direct microlaryngoscopy with CO2 laser excision, injection of Kenalog, and application of mitomycin C for residual fibrosis. 

## Discussion

On review of the literature, there have been only a few case reports of IgG4-RD involvement in larynx and trachea. Specifically, there have been four cases reported to our knowledge of subglottic involvement, as seen in our patient [3-6]. A handful of IgG-RD cases have involvement of the supraglottic region [6-8]. Overall, subglottic involvement appears to be a rare manifestation of IgG4-RD. Given that tracheal and laryngeal involvement can develop insidiously and progress to critical airway obstruction, this case highlights the importance of keeping IgG4-RD in mind when diagnosing and treating upper airway stenosis, especially in patients who become refractory to local treatments. Additionally, the patient had undergone multiple biopsies showing indeterminate results, and ultimately, a larger excisional biopsy was necessary to obtain a more confident diagnosis of IgG4-RD. Biopsies are the gold standard for diagnosis; however, the process of obtaining one is far from perfect. It is important to keep in mind that various factors can influence the biopsy, and false negatives can occur. If the clinical picture lends strong suspicion for IgG4-RD, then biopsy should be repeated to provide more definitive results. 

The treatment of IgG4-RD can be multimodal depending on the area involved [9]. The first line systemic treatment for IgG4-RD is glucocorticoids. Steroid-sparing agents such as rituximab can be employed as second-line options if the disease is refractory to steroids or if the steroids cannot be weaned to a dose that mitigates long-term side effects. Other immunosuppressive agents that can be considered include azathioprine and mycophenolate mofetil but are known to be only mildly effective [10-12]. In our case, local treatment with steroid injections was initially employed to suppress her disease while the diagnosis was being made. It is important to note that mature fibrosis may not respond to systemic immunosuppression and still requires surgical treatment [13]. The treatment of the patient can be multifaceted when the airway is involved; nevertheless, systemic immunosuppression is the mainstay of treatment to bring the disease into remission. 

## Conclusions

While laryngotracheal involvement in IgG4-RD is rare, our knowledge of this entity is in its infancy, and, thus, it may be under-recognized. It took nearly three years of follow-up and three biopsies to confidently characterize this patient’s disease. This case exemplifies the need for ongoing follow-up in patients with suspicion of systemic disease. This is particularly true if the presentation is in a location in which it is challenging to obtain a large tissue sample.
